# Cortical Ribbon and Crossed Cerebellar Diaschisis in Subclinical Status Epilepticus: A Case Report

**DOI:** 10.7759/cureus.36900

**Published:** 2023-03-30

**Authors:** Mohammad Abu-Abaa, Omar Jumaah, Aliaa Mousa, Ali Abdulsahib

**Affiliations:** 1 Internal Medicine, Capital Health Regional Medical Center, Trenton, USA

**Keywords:** crossed cerebella diaschisis, status epilepticus, seizure, gyriform dissuison restriction, cortical ribbon

## Abstract

Cortical ribbon is an uncommon finding that is characteristic of Creutzfeldt-Jakob disease but has a broad differential diagnosis. On the other hand, crossed cerebellar diaschisis is also an uncommon finding in brain magnetic resonance imaging (MRI). Herein, we are describing an 88-year-old male patient with dementia, ambulatory dysfunction, and frequent falls who presented with acute on chronic right-sided subdural hemorrhage that was discovered after an episode of seizure. Although the subdural hemorrhage was associated with mild midline shift and lateral ventricle compression, no surgical drainage was attempted, and only middle meningeal artery embolization was pursued. Lack of further evidence of seizure and clinical stability prompted discharge. However, he was soon re-admitted for left-sided focal seizure that failed multiple antiepileptic medications and evolved into status epilepticus. MRI brain showed evidence of both cortical ribbon as well as crossed cerebellar diaschisis. No evidence of infection or autoimmune inflammation was found with continuous mental status deterioration. Code status was changed by his family, and comfort care was pursued. This case is not only interesting because of the rarity of both cortical ribbon and crossed cerebellar diaschisis, but this case helps to remind clinicians of the relationship between these findings and seizure/status epilepticus.

## Introduction

Cortical ribbon, also referred to as gyriform cortical restricted diffusion (GCRD), refers to hyperintensity on diffusion-weighted imaging (DWI) and corresponding hypointensity of apparent diffusion coefficient (ADC) sequences of magnetic resonance (MRI). The most common etiology is cytotoxic edema secondary to vascular occlusion and restricted diffusion [1]. However, this can also be seen in several other neurological conditions. It is usually the result of anaerobic metabolism, lactic acidosis, and failure of the sodium/potassium ATPase pump resulting in cytotoxic edema [2]. Although cortical ribbon has been described as a marker for rapid neuronal death, neuronal death may not be present as GCRD can be reversible in certain neurological disorders such as seizure and hyperammonemia [1]. 

## Case presentation

**Figure 1:**
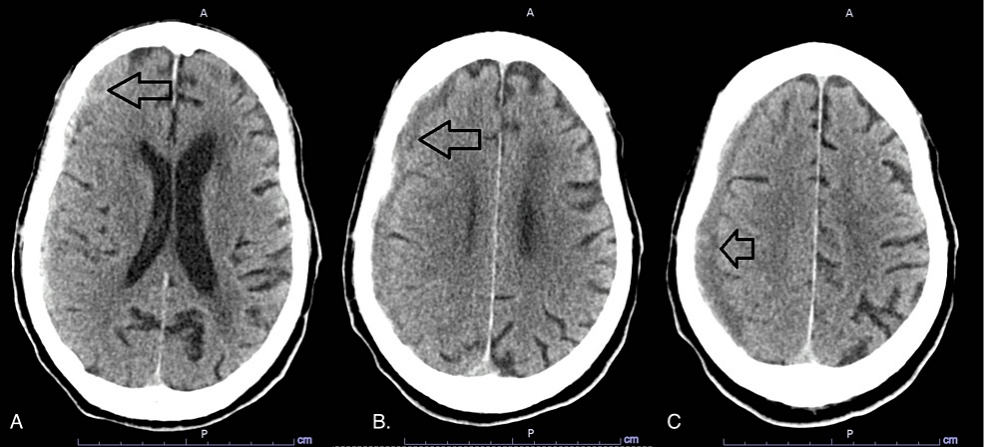
CT scan of the head Computed tomography (CT) scan of the head shows right-sided subdural hemorrhages with different densities at different levels (arrows in A, B, and C)

An 88-year-old male patient presented to the emergency department (ED) for an episode of loss of consciousness with urinary incontinence but without abnormal body movement. Past medical history was significant for dementia on memantine, hypertension on lisinopril, and ambulatory dysfunction with previous falls with T5 compression fracture. Vital signs included a temperature of 36.5 degrees Celsius, heart rate of 88 beats per minute, respiratory rate of 18 cycles per minute, blood pressure of 140/75 mmHg, and SpO2 of 98% on room air. On physical examination, he was lethargic and slow to respond, had mildly slurred speech, oriented to self and place, right gaze preference, bilateral equal +2 deep tendon reflexes in all four extremities, muscle power of 5/5 on all four extremities, intact light sensation grossly, normal tone. Computed tomography (CT) head showed an 11 mm mixed density right-sided subdural hematoma (SDH) with mild mass effect on the underlying cortex and compression of the lateral ventricle and 4 mm midline shift (Figure 1). He received levetiracetam 500 mg twice daily for seizure prophylaxis and was placed on continuous electroencephalography (EEG) monitoring.

**Figure 2:**
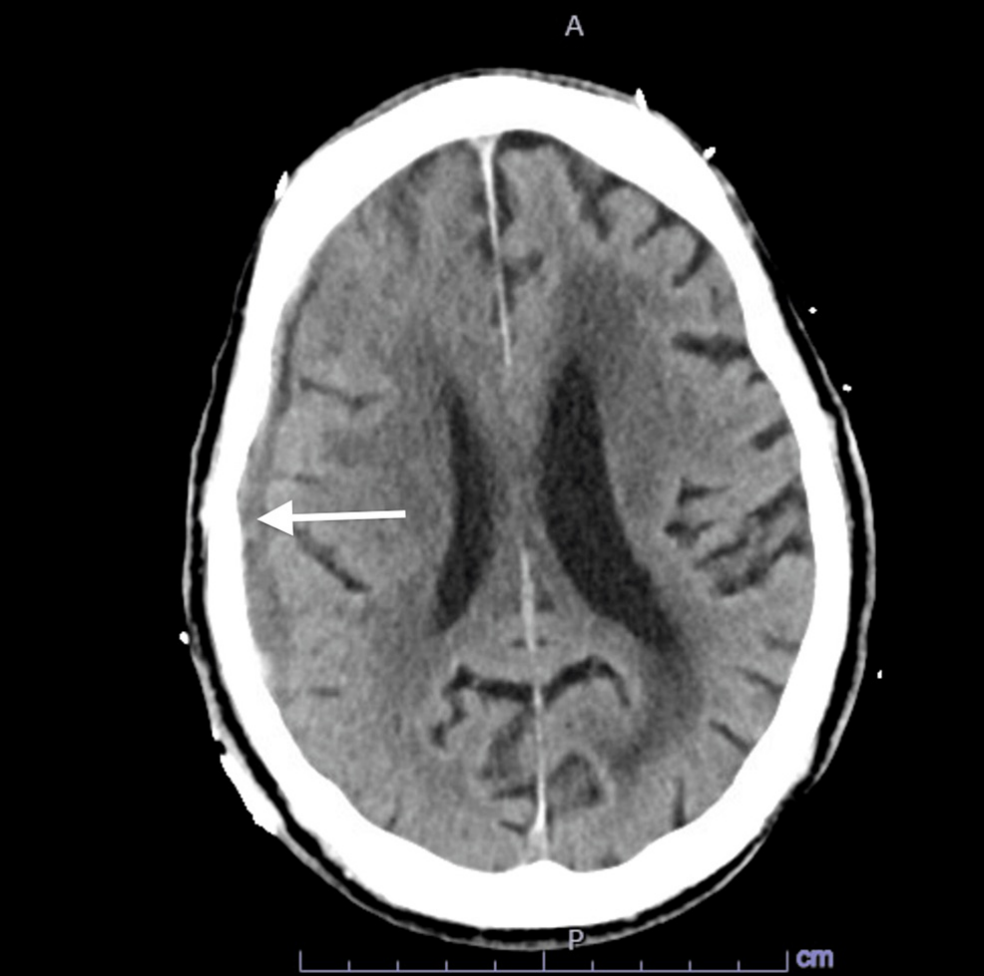
CT scan of the head Computed tomography (CT) scan of the head shows a relatively stable size of the right-sided subdural hemorrhage (arrow)

His orientation and alertness improved gradually after admission. EEG over three days showed moderate generalized slowing with no epileptiform discharges, and a repeat CT head showed stability of SDH (Figure 2). Diagnostic cerebral angiogram with successful embolization of right distal middle meningeal artery, frontal and parietal branches. He remained clinically stable and was discharged eight days after his admission. He was readmitted one day after discharge for a change in mental status. In the ED, he was awake, spontaneously opening his eyes, symmetrical face, not answering questions, not following commands, intermittently staring off to the left, and had jerky movements of the left upper and lower extremities. A repeat CT head showed no change in SDH as compared to imaging three days prior. Seizure was suspected, and the levetiracetam dose was increased to 1500 mg twice daily. An EEG showed slow lateralized periodic discharges (LPDs) over the right frontotemporal region at 0.75-1 Hz with moderate generalized slowing. This was suggestive of a highly epileptogenic focus in the right frontotemporal region. Twenty-four hours later, he remained encephalopathic, but gaze deviation was no longer evident. Abnormal movement involved only the left lower extremity and was time locked with the periodic discharges on EEG. The dose of levetiracetam was increased to 2000 mg twice daily with the addition of lacosamide 200 mg twice daily. No further changes were detectable on the CT head. 

**Figure 3:**
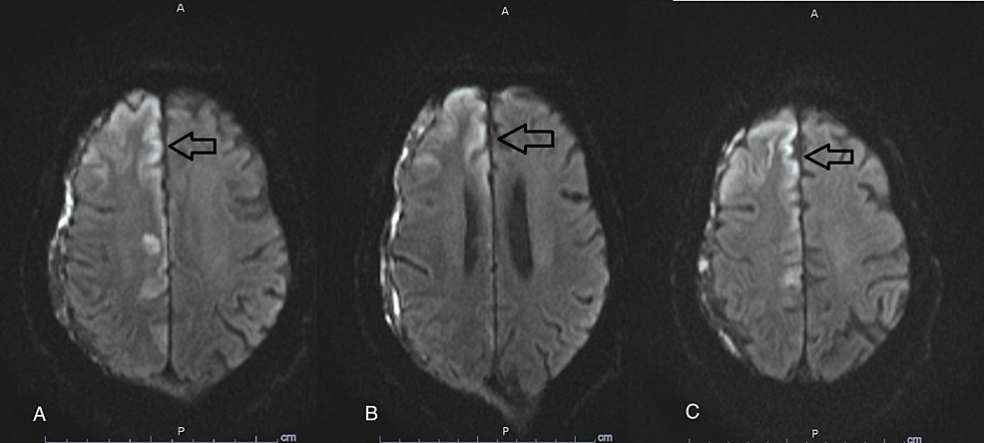
MRI diffusion trace sequence Magnetic resonance imaging (MRI) of the brain diffusion trace sequence showing evidence of diffusion restriction/cortical ribbon affecting the right frontal and paramedical regions (arrows in A, B, and C)

**Figure 4:**
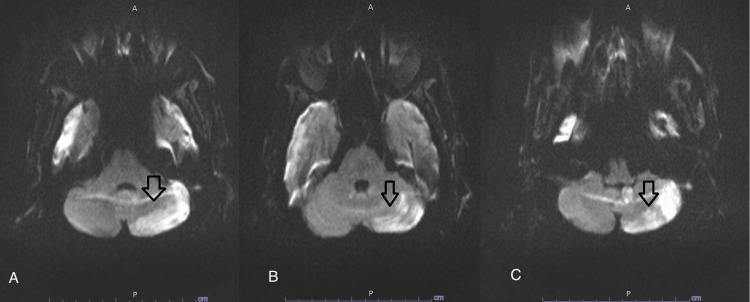
MRI diffusion trace sequence of the cerebellum Magnetic resonance imaging (MRI) brain diffusion trace sequence showing evidence of diffusion restriction affecting the left cerebella ray hemisphere (arrows in A, B, and C)

**Figure 5:**
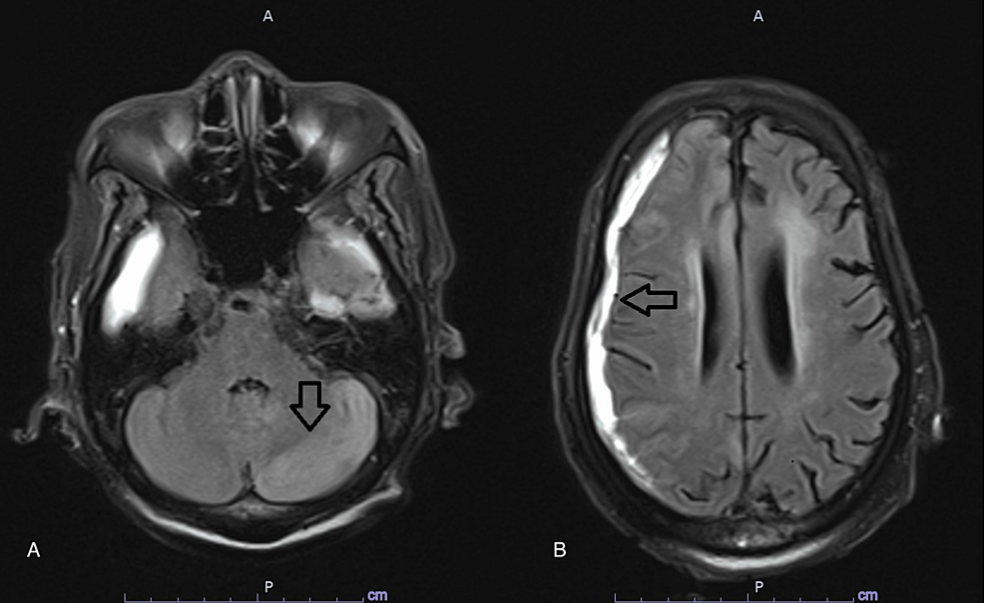
MRI FLAIR sequence Magnetic resonance imaging (MRI) brain fluid-attenuated inversion recovery (FLAIR) sequence showing left-sided cerebellar hyperintensity (arrow in A) and right-sided subdural hemorrhage (arrow in B)

Twenty-four hours later, he remained encephalopathic, not withdrawing to pain, with sluggish bilateral pupils, no gaze deviation, and intermittent jerky movements affecting the left lower extremity. LPDs persisted as well and remained time-locked with left lower extremity movements. He was loaded with fosphenytoin along with other antiepileptic medications (AEM). Lack of further clinical improvement of his seizure, along with LPDs persistence, prompted discontinuation of fosphenytoin and loading with sodium valproate 500 mg twice daily. Magnetic resonance imaging (MRI) brain showed bilateral subdural hemorrhages with a 1-2 mm left-sided midline shift. It also showed cortical diffusion restriction in the anterior and paramedian right frontal lobe without mass effects, diffusion restriction in the right cingulate gyrus, and diffusion restriction in the left cerebellar hemisphere (Figures 3, 4, 5).

Mental status remained poor with a calculated Glasgow Coma Score of three with an eventual transition of LPDs to generalized periodic discharges (GPDs) at 1 Hz, suggestive of generalized cortical hyperexcitability. No aggressive intervention was pursued given his code status of do not resuscitate/do not intubate (DNR/DNI). Clobazam was added to other AEMs. Lumbar puncture (LP) and cerebrospinal fluid (CSF) analysis showed lymphocytic pleocytosis of 12 cells/mcl, with elevated protein of 146 mg/dl and elevated glucose of 109 mg/dl. CSF culture, as well as meningitis/encephalitis panel, were negative. Awaiting the autoimmune encephalitis panel, empiric treatment for possible autoimmune encephalitis was pursued, and the patient received five days of intravenous immunoglobulin (IVIG). However, the autoimmune encephalitis panel was non-reactive. In addition, no clinical improvement in his mental status was appreciated, even after tapering down antiepileptic medications. The goals of care were discussed with his family, and a decision to pursue comfort care was pursued.

## Discussion

Cortical ribbon is typically observed in patients with Creutzfeldt-Jakob disease (CJD) as hyperintensity along the cortical gyri on diffusion-weighted imaging (DWI) magnetic resonance imaging (MRI). This is more apparent and easier to observe on DWI than on a fluid-attenuated inversion recovery (FLAIR) MRI sequence, where it appears as hyperintensity [3]. Interestingly, these MRI changes can be seen up to 2.5 to three years prior to the clinical presentation of CJD [4]. Although it is non-specific for CJD, cortical ribboning has a higher diagnostic accuracy of CJD than serological tests, including protein 14-3-3, neuron-specific enolase, S100 beta, and total tau protein [5]. 

Cortical ribbon has been seen as a marker of rapid neuronal loss secondary to prion disease (CJD). The notion of its association with other etiologies has suggested it is a marker of neuronal injury rather than death. Other etiologies include hypoxic-ischemic encephalopathy; hypoglycemic encephalopathy; anemia; infections, including cerebritis, rabies, herpes encephalitis; seizure, especially status epilepticus; cerebral venous thrombosis; metabolic, including hyperammonemia and hepatic encephalopathy; reversible cerebral vasoconstriction syndrome; electrolyte and mitochondrial disorders, including mitochondrial myopathy, encephalopathy, lactic acidosis, and stroke-like episodes (MELAS) [6]. It can also be seen in Wernicke encephalopathy, hepatic encephalopathy, and autoimmune encephalitis, such as anti-NMDAR encephalitis [7]. It is worth to be reminded that CJD can present initially with seizure prior to other manifestations in up to three percent of cases, and types of seizure can include non-convulsive status epilepticus (NCSE), myoclonus, and epilepsia partialis continua (EPC) [8]. Therefore, cortical ribbon along with seizure without a clear etiology of the seizure should raise the possibility of CJD. 

Post and peri-ictal imagining changes on both functional and anatomical brain imaging have been described for many years. These have been reported to be seen both locally and remotely. These lesions are typically characterized by increased T2 intensity and diffusion restriction on the apparent diffusion coefficient (ADC) window. This likely represents cytotoxic edema rather than neuronal death [9]. These changes might be persistent or reversible and can sometimes lead to cortical gliosis [10]. These changes have been reported after single or recurrent seizures [11]. There is no estimated prevalence of cortical ribbon in seizure and status epilepticus, given the rarity of this observation. 

Both local and remote white matter changes can be seen on imaging in those with seizures, especially status epilepticus. Remote lesions have been seen in the splenium of the corpus callosum, hypothalamus, basal ganglia, and contralateral cerebellum, and this is known as crossed cerebellar diaschisis. Posterior leukoencephalopathy, and ipsilateral and contralateral diencephalic lesions have also been reported. The pathological mechanism of remote lesions, in this case, crossed cerebellar diaschisis, is largely unknown. The suggested mechanisms include local edema, antiepileptic drug toxicity, or micro vacuolization of myelin. The most acceptable theory is remote lesions secondary to epileptic activity per se with associated increased neuronal activity, metabolic and vascular changes, including local blood-brain barrier breakdown and increased tissue permeability [9]. 

Crossed cerebellar diaschisis (CCD) refers to depressed metabolism and blood flow to the cerebellum secondary to a contralateral supratentorial cerebral insult. Although the exact pathological mechanism is unknown, it is likely the result of the disruption of cortico-ponto-cerebellar pathways [12]. It has been shown to have prognostic significance and was reported in association with cerebral tumors, epilepsy, infarction, and encephalitis. In status epilepticus, the excessive excitatory impulse from the epileptic foci leads to contralateral cerebellar deactivation through the cortico-ponto-cerebellar pathway [13]. This has been usually described in positron emission tomography (PET) and single photon emission computed tomography (SPECT) but has been rarely seen on MRI [14]. 

## Conclusions

Cortical ribbon or gyriform cortical diffusion restriction is an uncommon finding. Although it is characteristic of CJD, it does have a broad differential diagnosis. Crossed cerebellar diaschisis is also an uncommon MRI finding that is associated with a contralateral cortical pathology. Both of these findings can be seen in patients with seizure/status epilepticus.
